# Levels of Heavy Metals in Adolescents Living in the Industrialised Area of Milazzo-Valle del Mela (Northern Sicily)

**DOI:** 10.1155/2014/326845

**Published:** 2014-09-23

**Authors:** Monica Interdonato, Alessandra Bitto, Gabriele Pizzino, Natasha Irrera, Giovanni Pallio, Anna Mecchio, Antonino Cuspilici, Letteria Minutoli, Domenica Altavilla, Francesco Squadrito

**Affiliations:** ^1^Department of Clinical and Experimental Medicine, Section of Pharmacology, University of Messina, Torre Biologica 5th Floor, c/o AOU Policlinico G. Martino, Via C. Valeria Gazzi, 98125 Messina, Italy; ^2^Assessorato Territorio ed Ambiente, Regione Sicilia, Via Ugo La Malfa, 90146 Palermo, Italy; ^3^Department of Pediatric, Gynaecological, Microbiological and Biomedical Sciences, University of Messina, 98125 Messina, Italy

## Abstract

In the Milazzo-Valle del Mela area, the presence of industrial plants and the oil refinery make local residents concerned for their health. For this reason, we evaluated the levels of heavy metals in 226 children aged 12–14 years, living in the 7 municipalities of the area. A control age-matched population (*n* = 29) living 45 km far from the industrial site was also enrolled. Arsenic, cadmium, chromium, mercury, nickel, and vanadium were analysed in 24 h urine samples, while lead concentration was evaluated in blood samples. A questionnaire regarding life style and risk perception was also administered. Adolescents from Milazzo-Valle del Mela had cadmium levels significantly higher compared to either controls  (*P* < 0.0001) or the reference values of the European Germany Environmental Survey (GerES-IV) and the American National Health and Nutrition Examination Survey (NHANES). Furthermore, children had higher perception of living in a high-risk environment. The present data, for the first time, clearly indicate that adolescents living in Milazzo-Valle del Mela have increased body concentration of cadmium, which may be harmful to human health. These results deserve particular attention by the local and regional government to initiate prevention programmes in this susceptible population.

## 1. Introduction 

In 1986, the Italian Environment Ministry has officially recognized a number of high-risk areas across Italy, and over the last two decades other areas have been added to the list. The current number of high-risk areas is 53, including 3 in Sicily, where past and current industrial activities caused environmental changes that may be harmful to human health. The Sicilian region has three contaminated sites: the areas of Milazzo-Valle del Mela, Augusta-Melilli-Priolo, and Gela.

A recent epidemiological study [1] in a cohort of subjects living in the Gabbia district (Valle del Mela area) showed that mortality rate and hospital discharge records for cancer were enhanced. In agreement with this finding, the analysis of the Epidemiological Observatory [2] for specific cancers showed statistically significant increases in the percentage of colon and rectum tumours and multiple myeloma. Furthermore, this analysis pointed out that female population showed an increased incidence of breast cancer, while male population had a significant increase in malignant cancer of the trachea, bronchi and lungs, central nervous system, and thyroid.

Very few studies have addressed the possible effects of chronic low environmental exposure to mixtures of heavy metals in the general population of industrialized countries, especially with regard to their possible interactions with organs and tissues. Furthermore, there is a definite paucity of data concerning children; this is a specific cause for concern: in fact, children are known to absorb metals more readily than adults and are particularly sensitive for biologic and developmental reasons [3].

Most of our knowledge concerning the health effects of toxic metals largely stems from studies conducted on populations with relatively high exposure, such as workers in industry or in heavily polluted environments. Only in the last years few studies concerning human biomonitoring (HBM) have addressed the possible effects of chronic low environmental exposure to mixtures of these metals in the general population of industrialized countries, especially that particularly susceptible such as adolescents.

Indeed, concerns have been raised that children may be more susceptible to toxic exposure than adults because they have proportionally more intake of food contaminants, active developmental processes, multiple exposure pathways, and susceptible sociobehavioral activities [4].

Looking at the HBM preexisting situation in Italy, the evaluation of the internal dose of metals in the general population is available only for a few geographical areas in Italy and only the Lazio region investigated children [5, 6]. Furthermore, no data is available for the Sicilian region. To overcome this lack of information, some countries derive reference values for the general adolescent population using data from surveys of large numbers of individuals, such as the German Environmental Survey (GerES IV) [7, 8] and the U.S. National Health and Nutrition Examination Survey [9].

In light of these observations, the primary goal of this paper was to perform a biomonitoring study in adolescents living in the Milazzo-Valle del Mela area to identify their exposure to arsenic, cadmium, chromium, lead, mercury, nickel, and vanadium: in fact, these heavy metals are typical emissions by oil refinery and thermal power plants that are localized nearby.

## 2. Materials and Methods

### 2.1. Study Design

We used the methodically based criteria for the application of Human Biomonitoring developed from the Human Commission of the Federal German Environmental Agency [10]. The working principles and procedures of the commission have been previously summarised by Ewers et al. [11].

**Figure 1:**
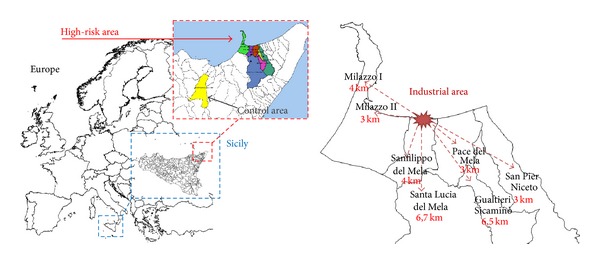
Geographical localization of the high-risk and control areas.

The study was performed between September 2012 and June 2013. Children (12–14 years) in the exposed group were recruited from those born and living in the area of Milazzo-Valle del Mela (including the following districts: Condrò, Gualtieri Sicaminò, Milazzo, Pace del Mela, San Filippo del Mela, Santa Lucia del Mela, and San Pier Niceto); the distance between the industrial and the residential districts was in a range between 1 and 7 km. Starting from December 2012 and up to January 2013, we met the parents in the different schools and, after an exhaustive explanation of the project, we obtained a written consent from the parents of all the subjects involved in the study. A total of 215 exposed children, 113 males and 102 females, aged 12–14 years, were enrolled. As control group, we enrolled 29 children (16 males and 13 females), aged 12–14 years and living 45 km far from the industrial plants in the Montalbano Elicona district (Figure 1).

Exclusion criteria for both exposed and controls were as follows: being of Sicilian origin, living in the selected area from at least 10 years, and absence of confounding diseases (i.e., diabetes, anaemia, etc.).

All children were provided with urine collection containers for 24-hour specimens and their parents were instructed for appropriate procedure and storage. Urine collection was performed 1 or 2 days before the medical visit and stored at 2–6°C to avoid any loss of sample or contamination. The 24-hour collection was done on Sunday, when the children were not at school. All medical visits were performed on Monday and Tuesday, afternoon, in the outpatient of Milazzo Hospital. As part of the visit, urine containers were collected and blood was withdrawn from a peripheral vein of the forearm. Furthermore, all children underwent a complete clinical evaluation. In addition, both parents and children were administered a multiple-choice questionnaire to assess their lifestyle, quality of life, and risk perception.

### 2.2. Sample Storage and Analysis

Following biological sample collection, the investigators delivered the samples to the Pharmacology and Toxicology Laboratory of the University of Messina. Urine total volume was recorded; samples (100 mL) were treated with 2 mL of 90% pure HNO_3_ and then stored at −20°C until analysis at the Laboratori Riuniti SCaRL (Catania, Italy). Blood samples were stored at −20°C until analysis.

### 2.3. Heavy Metals Analysis

Urine samples were analysed for the following heavy metals: arsenic, cadmium, chromium, mercury, nickel, and vanadium, and were determined to be blinded on coded samples. The Perkin-Elmer (Norwalk, CT) AAnalyst 300 atomic absorption spectrometer (AAS) with an automated turret, deuterium arc background correction (AA-BG), and HGA-800 graphite furnace was used for all analyses. The furnace was equipped with an AS-72 autosampler. Hollow cathode lamps were used as light sources. The blood used for lead analysis was collected according to the Italian Institute of Health Guidelines [12]. Lead in blood samples was analyzed with a Zeeman/5 100 PC atomic absorption spectrophotometer with HGA-600 graphite furnace and AS-60 autosampler (all from Perkin-Elmer). An Intensitron hollow cathode lead lamp (Perkin-Elmer) was the light source. When the results exceeded the reference value, repeated sampling was performed according to guidelines [13]. According to the IUPAC guideline [14], the reference value is defined within the 95% confidence interval of the 95th population percentile of the distribution of concentrations of a specific compound or element in a body fluid of a reference population [7, 11]. The results in the form of descriptive statistics were expressed in *μ*g/L for either blood or urine. In fact, the presentation of creatinine-based analytical data and reference values is not anymore recommended [13].

### 2.4. Statistical Analysis

Data were processed using the statistical software package SigmaStat version 11.0 for Windows. Results were expressed as median values or geometric means, if not stated differently. Data normality was tested with D'Agostino-Pearson normality test and differences between exposed and control groups were evaluated with Mann-Whitney *U* test; *P* < 0.05 was considered statistically significant.

The percentiles and maximum values are shown in the tables to help describe the sample distribution. The percentiles (P5, P25, P75, and P95) provided for each metal convey useful information about the upper distribution of levels in the population. The 95th percentile levels and appropriate confidence intervals were used for reference values proposal. Overall, the distributions of metals in blood and urine were described by the following statistical parameters: number of participants (*N*), percentiles (P25; P50; P75; P95), upper value (U-GM), lower value (L-GM), geometric mean (GM), and the 95% confidence interval for the geometric mean (CI-GM).

When the percentage of samples <LOD was too elevated (as for lead, mercury, nickel, arsenic, and vanadium), we transformed the data (0 for values <LOD and 1 for values >LOD) in order to compare the percentage of samples below/above the LOD for each metal between exposed and control groups, thus estimating the relative risk to exceed LOD, using Fisher's exact test.

The correlation between BMI and heavy metals exposure was assessed using Pearson's test. Each test was considered statistically significant if the *P* value was equal to or less than 0.05.

A post hoc power calculation revealed that the power of the study was 100% assuming urinary cadmium as the main variable. The test was performed according to Rosner [15], using the following formula: Power = *Ф*{−*Z*_1−*α*/2_ + Δ/√*σ*_1_^2^/*n*_1_ + *σ*_2_^2^/*n*_2_}, where *n*_1_ was the sample size for the group of control adolescents, *n*_2_ was the sample size for the group of exposed adolescents, Δ was the absolute difference between the means relative to the primary variable of our study (urinary cadmium concentration), *σ*_1_ and *σ*_2_ were the variances of such means in our two study groups (exposed and control, resp.), *α* was the probability of type I error (set to 0.05), *β* was the probability of type II error (set to 0.2), *Z* was the critical value for *α*, and Φ was the function converting a critical *Z* value to power.

## 3. Results

### 3.1. Biomonitoring Data

215 adolescents (113 males and 102 females), born and resident in the Milazzo-Valle del Mela area, were recruited. The general characteristics of the whole population are shown in Table 1. No differences were observed in age and body mass index (BMI). To determine whether age or BMI might contribute to body burden of each metal, correlation analysis was performed in the studied adolescents. No statistically significant correlation was observed (results not shown).

Table 2 represents the limit of detection (LOD) of the analytical method used for determining heavy metal levels in biological matrix samples and the percentage number of samples below the limit. Due to the large amount of samples under LOD for arsenic, lead, mercury, nickel, and vanadium (Table 2), the data relative to these elements were excluded from descriptive statistics.

Table 3 shows heavy metals concentration determined in biological matrices of the exposed and control groups. Data are expressed as geometric means and 95% confidence intervals and were subdivided in percentiles from P5 to P95.

The concentration of each analyzed metal was compared between exposed and control groups (Table 4). Cadmium levels were significantly higher in exposed adolescents than in control ones (0.55 *μ*g/L versus 0.26 *μ*g/L, resp.; *P* < 0.0001; Table 4). We also observed that cadmium levels were higher in males than in females (*P* = 0.0009; Table 4) in control group.

**Figure 2:**
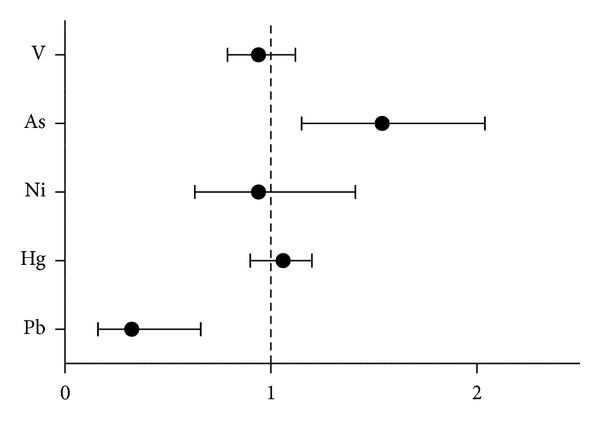
The graph represents the relative risk of having urinary/blood heavy metals levels above the LOD in exposed children compared to controls.

Analyzing the number of samples over-LOD for arsenic, lead, mercury, nickel, and vanadium, we found a significant increased relative risk (>1.54-fold; CI: 1.15–2.04; *P* = 0.01) to be exposed to arsenic (Figure 2) for exposed adolescents compared with controls; furthermore, the same analysis revealed that the control subjects were 3-fold more at risk of Pb exposure than those in the exposed group (Figure 2; relative risk: 3.07; CI: 1.49–6.32; *P* < 0.0001).

Heavy metals levels of both exposed and control children were compared with the reference values for not exposed adolescents as stated by previously published surveys (Table 5). Cadmium levels of exposed population were significantly augmented when compared with the reference values (Table 5).

Previous studies have investigated heavy metals exposure in adolescents living in other exposed areas of Europe; thus comparing our results to those, it was observed that cadmium values of adolescents living in our exposed area were higher than those of Czech and Spanish ones and similar to those of Polish ones (Table 6).

### 3.2. General Habits and Risk Perception Questionnaire

We administered a questionnaire to the adolescents and their parents for the identification of modifying elements, including socioeconomic and lifestyle ones which could provide important information on susceptibility factors.  Table 7 shows the most relevant answers provided by either exposed group or control group regarding quality of life, risk perception, and food habits. From the obtained answers, it is possible to observe that none of the enrolled kids stated to be a smoker. Overall, adolescents living in the exposed area and their parents had higher perception of living in a high-risk environment.

## 4. Discussion

Environmental pollution by heavy metals in industrialized countries is the consequence of present or past emissions by petrochemicals industries. Unfortunately, few stringent measures and controls have been put into place and, as a consequence, high levels of these pollutants still persist in the soils and sediments causing concerns for the food chain integrity and unwanted consequences for the populations living in those areas.

In the present study, we evaluated heavy metals exposure in the adolescent population, resident in the Milazzo-Valle del Mela area, identified as a high-risk area by local authorities. A total of 215 adolescents aged 12–14 years were recruited in the seven schools located in the municipalities of Condrò, Gualtieri Sicaminò, Milazzo, Pace del Mela, San Filippo del Mela, Santa Lucia del Mela, and San Pier Niceto. An age-matched control group resident in a rural area of Sicily was also studied. Adolescents represent an essentially important population sample: in fact, exposure to heavy metals from the early childhood could have a negative impact on the developmental status. In addition, this population has not yet too many complicating influences, such as occupational exposure, moving house, which may cause variability in the data evaluation and interpretation.

The first most relevant result emerging from our survey is the presence of high levels of urinary cadmium in exposed adolescents compared with control ones, having levels within the range of the reference values [7, 9]. As a matter of fact, there are several industrial plants and metal-work-related factories, currently operating in the area, that use cadmium compounds as stabilizers for PVC products, color pigments, alloys, and rechargeable nickel cadmium batteries. In addition, the thermal power plant and metallurgical industries, still active and located in the area, use cadmium as an anticorrosion agent (cadmiation). In the present study, we identified gender difference in the urinary levels of cadmium in the exposed group: this may reflect either a different tissue accumulation or absorption in the two sexes, as already reported [16]. Airborne cadmium could be readily absorbed by plants and taken up through the roots to edible leaves, fruits, and seeds [17, 18], and this might also explain the elevated values observed in the adolescents: in fact, results from the questionnaire show that 67% of adolescents eat fruits and vegetables grown in their own garden. All these observations, taken together, led us to hypothesize that the elevated urinary levels of this heavy metal are due to a high burden of pollutants in both the ecosystem and the food chain.

The urinary cadmium concentration is mainly influenced by the body burden and is proportional to the concentration in the kidneys. In general, nonsmokers have urinary cadmium concentrations of 0.02–0.7 *μ*g/L, and their cadmium levels slowly increase with age together with the accumulation of cadmium in the kidney [19]. Therefore, elevated urinary cadmium excretion is a read-out of high intake of cadmium and also of elevated gastrointestinal absorption of the heavy metal in children, as recently pointed out by Kippler et al. [20]. However, the increased levels of cadmium of exposed adolescents compared with controls do not allow drawing any final conclusion about the health risk of the population living in the Milazzo-Valle del Mela area.

As a matter of fact, the German Human Biomonitoring Commission recommends the use of two different kinds of criteria to assess exposure: reference values and HBM values. The reference values indicate the upper margin of background exposure to a given pollutant in a given population at a given time [7]. By contrast, HBM values derive two different kinds of values: HBM I represents the concentration of a substance in human biological material below which there is no risk for adverse health effects and, consequently, no need for action; HBM II represents the concentration above which there is an increased risk for adverse health effects. Adverse health effects should be considered for concentrations in the range between HBM I and HBM II. The geometric mean of the urinary cadmium levels in our exposed adolescents was higher than the specific HBM I value (0.50 *μ*g/L) settled by German Human Biomonitoring Commission [8] and fell in the range between HBM I and HBM II (2 *μ*g/L). In another study, called DEMOCOPHES, a pan-European HBM project involving 120 mother and child (6–11 years) couples, cadmium levels were found higher in younger children, suggesting that the prepubertal age is even more sensitive to metals' accumulation [21]; given the different age-range, it is difficult to compare those results with our study.

Intense industrialization and other activities have led to the global occurrence of soluble chromium, which is readily leached from soil to ground water in concentrations above permissible levels. This naturally occurring element was found elevated in either exposed or control adolescents when compared with reference values [22]. This could be due to nonanthropogenic contamination of drinking water: in fact, most of the local mineral water has shown an elevated content of this metal (up to 50 *μ*g/L) during routine checks by the Sicilian Environmental Protection Agency (ARPA, Sicilia) [23]. Besides, it should be pointed out that we measured total chromium but not its hexavalent form that more correlates with an increased healthy risk. Finally, there is no survey showing reference value for this specific heavy metal in adolescents; of the previous surveys, only one carried out in a cohort of Spanish adolescents evaluated urinary chromium, demonstrating lower levels compared to the present study [24]. Therefore, our present data represent a warning for the local, regional, and national authorities that should undertake immediate action to reduce exposure to cadmium and chromium.

Surprisingly, we found higher blood lead levels in the control population than in exposed adolescents: this unexpected finding is likely linked to high levels of lead water pipes that have not yet been replaced in the rural houses. Another explanation could be the higher frequency of smoking parents as suggested by the results of our life style and habits questionnaire.

Additionally, adolescents living in Milazzo-Valle del Mela area had an increased risk of being more exposed to arsenic than those living 45 km away from the industrial site; this mainly reflects the frequency of fish consumption, in agreement with results of questionnaires, where it appears that the 40% of exposed adolescents (versus the 14% of controls) eat fish twice a week. Furthermore, the Sicilian Environmental Agency Report (ARPA, Sicilia) has detected overruns for arsenic in groundwater in the Milazzo-Valle del Mela area [23]. However, arsenic urine levels of the adolescents living in the industrial area were below the reference values [22, 25].

Mercury urinary levels were above reference value, in exposed children; however, the high percentage of samples below the limit of quantification makes the comparison with other studies particularly difficult. Besides the industrial emission, mercury levels are most likely due to the large use of dental amalgam fillings, and methylmercury exposure derives also from fish consumption [26].

## 5. Conclusions

In conclusion, the present biomonitoring study clearly shows that adolescents living in the Milazzo-Valle del Mela area have increased urinary levels of cadmium which requires an action to reduce human exposure. Besides, this study will serve as a basis for the health risk assessment and management process to favour the implementation of preventive measures to control emissions from the industrial plants of the area.

## Figures and Tables

**Table 1:** General characteristics of the studied population.

Characteristics	Milazzo-Valle del Mela	Montalbano Elicona
Female : male	102 : 113	13 : 16
Age (mean years ± SD)	13.32 ± 0.60	13.46 ± 0.69
BMI (mean ± SD)	21.21 ± 4.49	21.44 ± 4.87

**Table 2:** Limits of detection of the analytical method (LOD) and percentage number of samples below the LOD for each metal.

Heavy metals *µ*g/L	Pb	Cd	Cr	Hg	Ni	As	V
MATRIX	Blood	Urine	Urine	Urine	Urine	Urine	Urine
LOD	5	0.1	0.2	1	0.1	2	0.1
%<LOD exposed adolescents	65%	2%	11%	89%	52%	43%	85%
%<LOD control adolescents	21%	0%	0%	96%	48%	69%	83%

**Table 3:** Concentrations of metals in biological fluids of exposed (*n* = 215) and control adolescents (*n* = 29).

Heavy metals (*µ*g/L)	Matrix	*n*.	P5	P25	P75	P95	Median	GM	Low-CI	Upper-CI
Cadmium	Urine	210	0.20	0.30	1.00	1.42	**0.60**	**0.55**	0.50	0.61
Cadmium	Urine	29	0.10	0.20	0.30	0.50	**0.30**	**0.26**	0.22	0.30
Chromium	Urine	191	0.10	0.80	2.00	5.94	**1.20**	**1.18**	1.01	1.36
Chromium	Urine	29	0.60	1.00	1.70	3.80	**1.20**	**1.25**	1.05	1.48

**Table 4:** Concentrations of metals in adolescents analyzed.

Heavy metals	Exposed group		Control group		*P* value
	*n*.	*µ*g/L	*n*.	*µ*g/L	
Cadmium	210	0.55	29	0.26	<0.0001
Male	111	0.58	16	0.32	
Female	99	0.54	13	0.20	
*P* value male versus female	n. s.		0.0009		
Chromium	191	1.18	29	1.25	n.s.
Male	102	1.31	16	1.36	
Female	89	1.07	13	1.12	
*P* value male versus female	n. s.		n. s.		

Data are expressed as geometric means (GM).

**Table 5:** Concentration of metals in adolescents reported in other surveys.

Heavy metals (*µ*g/L)	Present study		Reference survey				
	Exposed	Controls	GerES IV Report [22]	NHANES (CDC) Report [27]	Canadian Health Measures Survey Cycle [28]	Czech Republic Survey [29]	RV
Cadmium	0.58	0.26	0.08	0.08	0.27	0.80	0.2
Chromium	1.18	1.25	—	—	—	—	0.59

Data are geometric means (GM). All metals were evaluated in urine.

**Table 6:** Geometric mean concentration (*μ*g/L) of metals in adolescents living in Milazzo-Valle del Mela and Montalbano Elicona areas compared with other studies.

	Present study				France [30]				Poland [30]				Czech Republic [29, 30]				Spain [24]	
	Exposed		Controls		Exposed		Not exposed		Exposed		Not exposed		Exposed		Not exposed		Exposed	Not exposed
	F	M	F	M	F	M	F	M	F	M	F	M	F	M	F	M		
Cadmium	0.54	0.58	0.20	0.32	1.07	1.15	0.91	1.02	0.56	0.56	0.45	0.44	0.25	0.24	0.22	0.22	0.35	0.49
Chromium	1.31	1.07	1.12	1.36	—	—	—	—	—	—	—	—	—	—	—	—	0.39	0.37

**Table 7:** Quality of life and risk perception questionnaire administered to either parents or adolescents involved in the study. Results are expressed as percentage.

	Milazzo-Valle del Mela	Montalbano Elicona
**Parental questionnaire**		
How do you consider your social/working life?		
Normal	31%	69%
I have some concerns	60%	26%
I have a lot of concerns that limit my social/working life	8%	5%
I feel very worried	1%	0%
Which is the main source of pollution, in your area?		
Air and water	69%	24%
Food products	21%	0%
Sea products	7%	7%
All of the above	3%	69%
What do you think about the general environmental conditions of the Milazzo-Valle del Mela area?		
Great conditions, the wind blows away all contaminants	0%	17%
Good conditions, however sometimes there is a bad smell	7%	3%
I think it is risky only close to the industrial plants	5%	11%
The whole area is very polluted	88%	69%
Do you smoke?		
No, I do not	80%	59%
Less than 10 per day	10%	7%
Up to 20 per day	9%	27%
More than 20 per day	1%	7%
Do you buy fruit and vegetables from the local market?		
Yes, I prefer fresh products	20%	7%
Yes, however sometimes I go to the grocery stores	43%	48%
No, I don't	13%	14%
I grow my own vegetable garden	24%	31%
How many times a week do you eat fish?		
Once a week	47%	14%
Twice a week	27%	48%
One-two times a month	24%	38%
I do not eat fish	2%	0%
Do you use tap water for food purposes?		
Yes, I drink it everyday	8%	72%
Yes, but only for cooking	35%	25%
No, I do not	57%	3%

**Children questionnaire**		
Do you like the place where you live?		
Yes, a lot	25%	61%
Yes, but I would rather stay in a less polluted environment	58%	7%
Not much	13%	29%
No, at all	4%	3%
Do you think of living in a polluted area?		
Yes, my parents always complain about it	54%	7%
Yes, because I live downtown and there are a lot of vehicles	27%	18%
No, I live in outside of the town	19%	75%
What is the main source of pollution in your opinion?		
Industrial plants	91%	62%
Urban pollution from vehicles	6%	21%
I do not know	3%	17%
How is your house?		
It is outside of the town with a back yard	39%	50%
It is a condominium outside of the town	25%	39%
It is a condominium in downtown	36%	11%
How many hours a day do you spend playing outside?		
I never play outside	12%	6%
Maximum 1 hour, I rather prefer to watch TV	9%	2%
I spend most of my day outside only during spring/summer	61%	70%
I spend most of my afternoons outside in the garden	18%	22%
Do you have a vegetable garden?		
Yes, at home or at my grandparent's	67%	29%
No, but our neighbours have and sometimes give us some fresh vegetables	7%	57%
No, we buy only from the grocery store	26%	14%
How many times a week do you eat fish?		
I do not like fish	8%	21%
One-two times a month	19%	31%
Twice a week	33%	34%
Once a week	40%	14%
Do your parents let you drink tap water?		
Yes, I drink it everyday	3%	70%
No, we use it only for cooking	65%	28%
No, never	32%	2%
Do you smoke?		
No, I do not	100%	100%
Less than 10 per day	0%	0%
Up to 20 per day	0%	0%
More than 20 per day	0%	0%
